# Specificity and sensitivity of the fixed-point test for binary mixture distributions

**DOI:** 10.3758/s13428-023-02244-9

**Published:** 2023-11-13

**Authors:** Joaquina Couto, Maël Lebreton, Leendert van Maanen

**Affiliations:** 1 Department of Psychology, University of Amsterdam, Amsterdam, Netherlands; 2 Department of Experimental Psychology, Utrecht University, Utrecht, Netherlands; 3 Swiss Center for Affective Sciences, University of Geneva, Geneva, Switzerland; 4 Paris School of Economics, Paris, France

**Keywords:** Binary mixture data, Fixed-point property, Empirical validation

## Abstract

When two cognitive processes contribute to a behavioral output—each process producing a specific distribution of the behavioral variable of interest—and when the mixture proportion of these two processes varies as a function of an experimental condition, a common density point should be present in the observed distributions of the data across said conditions. In principle, one can statistically test for the presence (or absence) of a fixed point in experimental data to provide evidence in favor of (or against) the presence of a mixture of processes, whose proportions are affected by an experimental manipulation. In this paper, we provide an empirical diagnostic of this test to detect a mixture of processes. We do so using resampling of real experimental data under different scenarios, which mimic variations in the experimental design suspected to affect the sensitivity and specificity of the fixed-point test (i.e., mixture proportion, time on task, and sample size). Resampling such scenarios with real data allows us to preserve important features of data which are typically observed in real experiments while maintaining tight control over the properties of the resampled scenarios. This is of particular relevance considering such stringent assumptions underlying the fixed-point test. With this paper, we ultimately aim at validating the fixed-point property of binary mixture data and at providing some performance metrics to researchers aiming at testing the fixed-point property on their experimental data.

## Introduction

One core objective of cognitive and behavioral sciences is to identify and decipher the hidden, internal variables and operations used by individuals to solve specific problems or tasks at hand. For example, in economic decision-making under risk, the dominant theories assume that individuals compute a subjective expected value for each available option, and choose the option with the highest value (McFadden, 1999; Rabin, 1998; Rangel et al., 2008). However, most cognitive tasks can be solved—more or less optimally—through a variety of strategies, implying different sets of operations and variables (Gigerenzer & Gaissmaier, 2011; Vlaev et al., 2011). Competing theories inspired by bounded rationality principles have therefore proposed that individuals rely on heuristics—i.e., simple deterministic rules—to make their choices (Brandstätter et al., 2006; Glöckner & Betsch, 2008; Payne et al., 1988). Usually, the debate about the latent variables and operations that are involved in economic decision-making under risk revolves around which of these theories best explains the overall, complex picture of choices produced by participants over one or several experiments. Ultimately, however, individuals could not only use one dominant strategy, but alternate between different strategies—i.e., use a mixture of strategies. Using different strategies to perform a specific problem or task has indeed been reported in a wide variety of experimental tasks, not only in adaptive decision-making (Collins & Frank, 2013; Domenech & Koechlin, 2015), but also in economic decision-making (Couto et al., 2020; Lopez-Persem et al., 2016), perceptual decision-making (Ashwood et al., 2022; Roy et al., 2021), language processing (Ramotowska, 2022), and arithmetic problem-solving (Groeneweg et al., 2021). Furthermore, individuals could alternate between strategies as an adaptation to changing task demands (Cohen et al., 2007) or as an exploration of the different strategies to perform the task (Knox et al., 2012). This more flexible view of different strategies generating human behavior is also endorsed by dual-process theories of cognition (Evans, 2003; Sloman, 1996). Accordingly, an increasing number of studies have acknowledged the importance of assessing whether a behavioral variable of interest is the product of one or several different strategies (Archambeau et al., 2022; Visser & Speekenbrink, 2014), and of deciphering experimental factors that would favor one strategy over another (Couto et al., 2020; Roy et al., 2021).

**Fig. 1:**
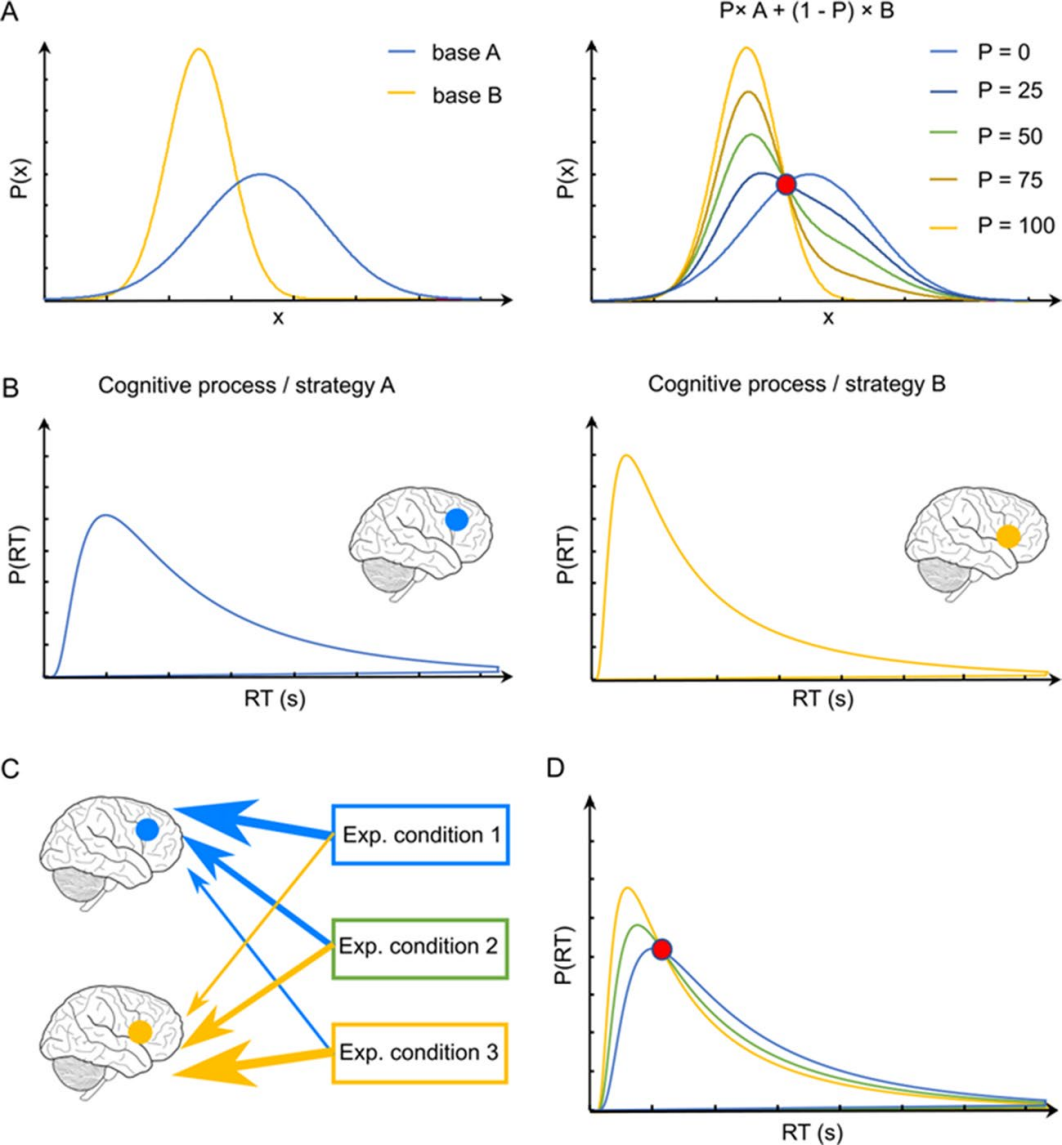
**A** Illustration of the fixed-point property in binary mixture data. The fixed-point property entails that any mixture of two base distributions (base A and B) cross at the same common density point, regardless of the mixture proportion, P (%). Densities with various mixture proportions of base A and B are displayed. The red dot indicates the fixed point. **B** Distributions of a measured behavioral variable (RTs) for which two different generative cognitive processes are hypothesized (strategy A and B). For illustrative purposes, the two strategies are displayed with different brain areas. **C** Illustration of the fixed-point property in experimental data for which the mixture of strategy A and B is manipulated with three experimental conditions. In condition 1, the mixture proportion of strategy A is P ≈ 100%, and the mixture proportion of strategy B is P ≈ 0%. In condition 2, the mixture proportion of both strategies is P = 50%. In condition 3, the mixture proportions of strategy A and B are the reciprocal of the mixture proportions in condition 1. **D** The observed RT distributions display the shared density point

In a series of recent papers, we described a method to identify the presence of a mixture of cognitive processes generating a behavioral variable—e.g., response times (RT) (Van Maanen et al., 2014, 2016). This so-called fixed-point property of mixture distributions entails that, independent of the mixture proportion, there will always be one probability density that is shared across all possible mixtures of the same two base distributions (Falmagne, 1968). This is illustrated in Fig. 1A, where distributions A and B are mixed with different proportions but all cross at the same—fixed—probability density. The presence of a fixed point can be tested on distributions of a measured behavioral variable for which two different generative cognitive processes are hypothesized and their mixture assumed to vary as a function of an experimental factor (Brown et al., 2006; Van Maanen et al., 2014). Consider an experiment where two processes jointly account for the final behavior (e.g., RTs, Fig. 1B) and the relative contribution—i.e., mixture proportion— of each process for the final behavior changes, depending on the experimental conditions (Fig. 1C). The fixed-point property entails that the observed distributions of a dependent variable for the different experimental conditions all cross at the same point (Fig. 1D). Because such a fixed point is extremely unlikely to be present in the data—i.e., only when the data come from a binary mixture is the experimental manipulation strong enough to affect the mixture proportions, and only the mixture proportions are affected and no other property of the data—observing such a property in the data would be strong evidence for a mixture of two strategies. Note that the fixed-point property does not require assumptions about the shape of the distributions; by extension, neither a mechanistic theory of the processes nor a model of the hidden cognitive variables and operations is required for testing the presence or absence of the mixture using the fixed-point property.

The procedure to statistically test for the presence or absence of the fixed-point property in experimental data (Van Maanen et al., 2014, 2016) involves four steps. In step 1, a first statistical test evaluates whether the data collected across the different conditions exhibit signatures of a behavioral change caused by the experimental manipulation. If there is no statistical difference between the distributions of the behavioral variable elicited across the different conditions, the fixed-point property cannot be properly tested (Van Maanen et al., 2016). In step 2, the distributions of the behavioral variable of interest are estimated using a Gaussian kernel-based density estimator for each participant and condition. This means that based on the collection of discrete data points, a smoothed histogram (i.e., a distribution, or density) is produced that summarizes and interpolates how the behavioral variable is distributed in each condition. In step 3, for each pair of experimental conditions, the point where the respective density approximations cross is computed. Thus, in an experiment featuring three conditions (Fig. 1C), there are three pairs of distributions (i.e., condition 1–condition 2, condition 2–condition 3, and condition 1–condition 3) and consequently three crossing points per participant (Fig. 1D). In step 4, a statistical analysis is performed to determine whether the three crossing points estimated from the empirical distributions of the participants are more likely to be sampled from a unique distribution—which is evidence in favor of a fixed point–or from statistically different distributions, which is evidence against a fixed point.

For this last step, the typical approach has been to compute a Bayes factor (BF) in favor of the presence of a fixed point using Bayesian analysis of variance (Rouder et al., 2012). A BF > 1 indicates that a fixed point is more likely to be present than to be absent, and a BF < 1 means that a fixed point is more likely absent than present. Using the four-step approach sketched above, we and others have found evidence for a mixture of processes in task-switching (Grange, 2016; Poboka et al., 2014; Van Maanen et al., 2014) and in economic decision-making (Couto et al., 2020), and evidence against mixtures in speed/accuracy trade-offs in decision-making (Katsimpokis et al., 2020; Van Maanen, 2016). In all these studies, the conclusion about the presence or absence of a fixed point (and hence a mixture of cognitive strategies) depended on the value of the BF alone. We never explicitly considered the probability of a false positive outcome (i.e., a lack of specificity of the method) or a false negative outcome (i.e., a lack of sensitivity of the method). The current paper assesses these probabilities through scenario analysis, consisting of resampling of real RT data.

### Scenario analysis

A common approach for determining the specificity and sensitivity of a test is to compute these under assumptions about the expected probability distribution of the data (Kuijpers et al., 2021; Molenaar et al., 2019). In the current paper, we develop a form of scenario analysis (Huss, 1988) to more closely reflect the true distribution in the data. In scenario analysis, a set of possible scenarios is determined, after which the distributions of possible outcomes are computed for each scenario, for example, through bootstrapping of a known data set. This approach has been widely applied in forecasting models, where (long-range) predictions are required under a fixed set of assumptions, such as in climate modeling (e.g., Xiao et al., 2019) and economic projections (e.g., Sandmann et al., 2021). In contrast, scenario analysis is less well known in the domains of psychological measurement, where the aim is to assess the validity of a test under various scenarios.

We systematically investigate the sensitivity and specificity of detecting a fixed point in different scenarios using signal detection theory (SDT, Green & Swets, 1966; Macmillan & Creelman, 2005). In order to assess the ability of the fixed-point property to make correct detections (true positives) and correct rejections (true negatives), we systematically resampled experimental data produced under two different strategy instructions so as to generate sets of three synthetic conditions. Thus, we could generate both positive controls, in which the RT data are actually produced by two strategies and whose mixture proportions varied across the three different synthetic conditions, and negative controls in which the RT data are also produced by two strategies but whose mixture proportions were fixed across the three different synthetic conditions. Importantly, the use of real RT data allows us to preserve important authentic features of the data which are only observed in real experiments, while maintaining tight control over the properties of the resampled data.

**Fig. 2:**
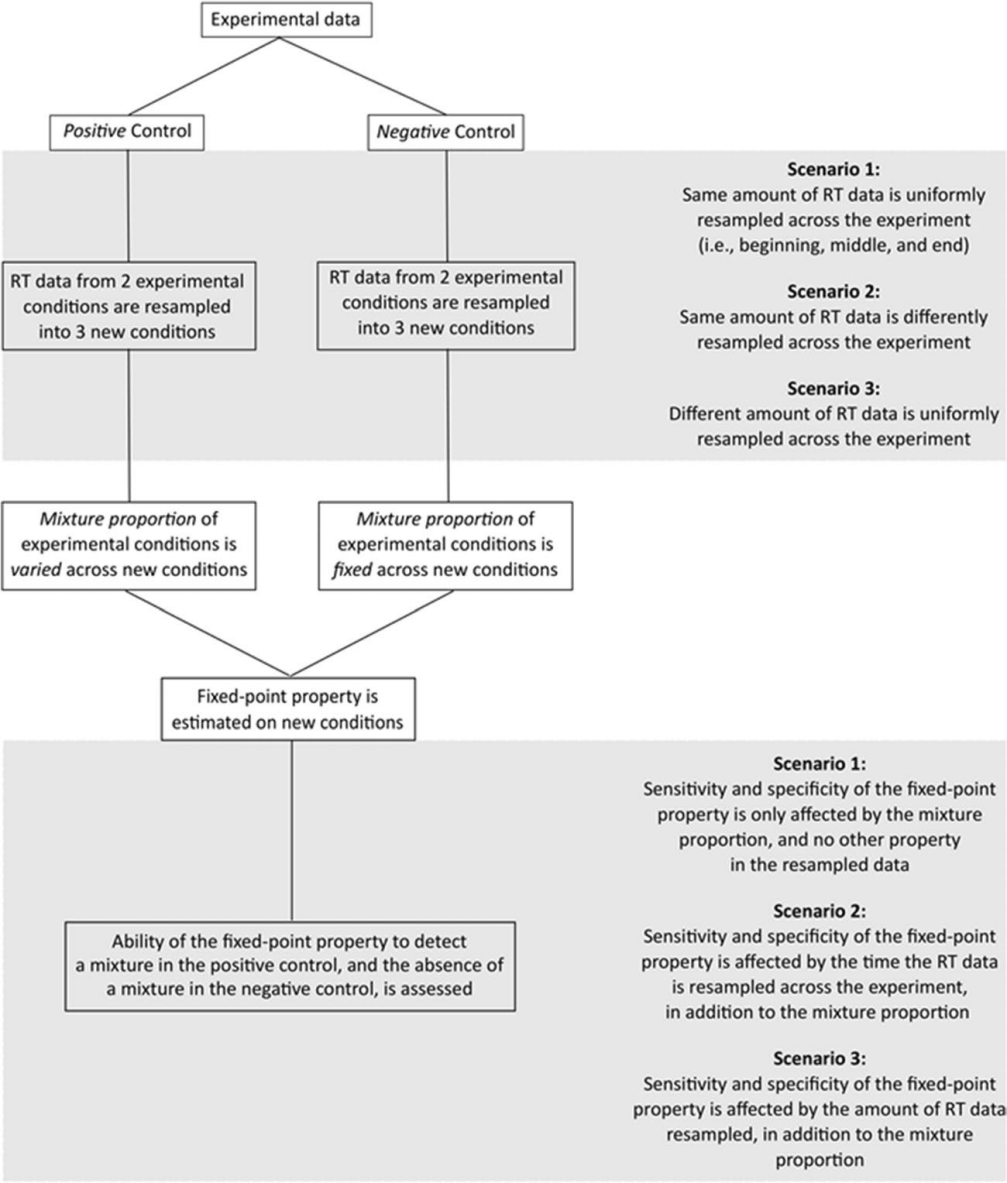
General procedure of resampled scenarios and their specifications. In all scenarios, RT data from two experimental conditions are resampled into three new conditions: in the positive control, the mixture proportion of the two experimental conditions varies across the three new conditions; in the negative control, the mixture proportion is fixed. The fixed-point property is estimated on the three new conditions, and the ability of the fixed-point property to detect a mixture in the positive control, as well as the absence of a mixture in the negative control, is assessed. In Scenario 1, only the mixture proportion varies; consequently, the sensitivity and specificity of the fixed-point test is affected only by the mixture proportion and no other property in the resampled data. In Scenarios 2 and 3, other properties of the resampled data vary—specifically, the probability that RT data are resampled from the beginning, middle, or end of the experiment in Scenario 2, and the amount of RT data resampled in Scenario 3. Consequently, the sensitivity and specificity of the fixed-point test are affected by the time the RT data are resampled across the experiment in Scenario 2 (i.e., time on task) and by the amount of RT data resampled in Scenario 3 (i.e., sample size), in addition to the mixture proportion

Leveraging this strategy, we investigated three types of scenarios. In Scenario 1, we assessed the ability of the fixed-point property to detect a mixture in the data, while varying how the mixture proportions changed in the different conditions of the positive control. Meanwhile, we also assessed the ability of the fixed-point property to detect the absence of a mixture when the mixture proportions did not change, i.e., in the different conditions of the negative control. This is a *reference scenario* in the sense that the sensitivity (captured in the positive control) and specificity (captured in the negative control) of the fixed-point test are affected only by the mixture proportion and no other properties in the data. In the second and third scenarios, the sensitivity and specificity of the fixed-point test are affected by other properties in the data, in addition to the mixture proportion—specifically, the duration of the experiment itself in Scenario 2 (what we call *time-on-task effects*) and the sample size of the experiment in Scenario 3 (what we call *sample size effects*). The general procedures for the resampled scenarios and their specifications are illustrated in Fig. 2.

## Methods

### Experimental data

In all resampled scenarios reported below, we reanalyzed RT data from a functional magnetic resonance imaging (fMRI) experiment. Data and scripts for performing bootstrapping for all scenarios are available at https://osf.io/9vs3y. The fMRI analyses and results are not reported here. In this experiment, participants were asked to choose between two lotteries that differed in the probability of winning a certain monetary outcome, as well as the value of the monetary outcome (Couto et al., 2020). In this setup, in the absence of explicit instructions, multiple strategies are available for participants to choose, such as computing and comparing the expected value of the options, or using a heuristic or rule of thumb (e.g., focus only on the probability of winning). In this study, to isolate the strategy that involves computing expected values, we explicitly instructed participants—and incentivized them accordingly—to either choose the option with the highest expected value or choose their preferred option, in a blocked design, resulting in different strategies.

### Participants

Participants were recruited from the laboratory's participant database (www.lab.uva.nl) of the University of Amsterdam. Participants provided all the necessary written forms before participating in the experiment (i.e., informed consent for the experiment itself and all the forms concerning safety which are required for fMRI experiments). Participants were rewarded with two research credits (RC) for their participation, with the possibility of a maximum monetary reward of €10, depending on two randomly chosen trials at the end of the experiment. All the experimental procedures followed the guidelines imposed (and approved) by the local Ethics Committee of the University of Amsterdam, Psychology Department (2019-PML-11490). The sample consisted of 48 participants, but four participants were excluded from the analyses for not completing the task, leaving 44 participants in the reported analyses (29 female, mean age = 21.1, SD = 2.5).

### Experimental design and procedure

**Fig. 3:**
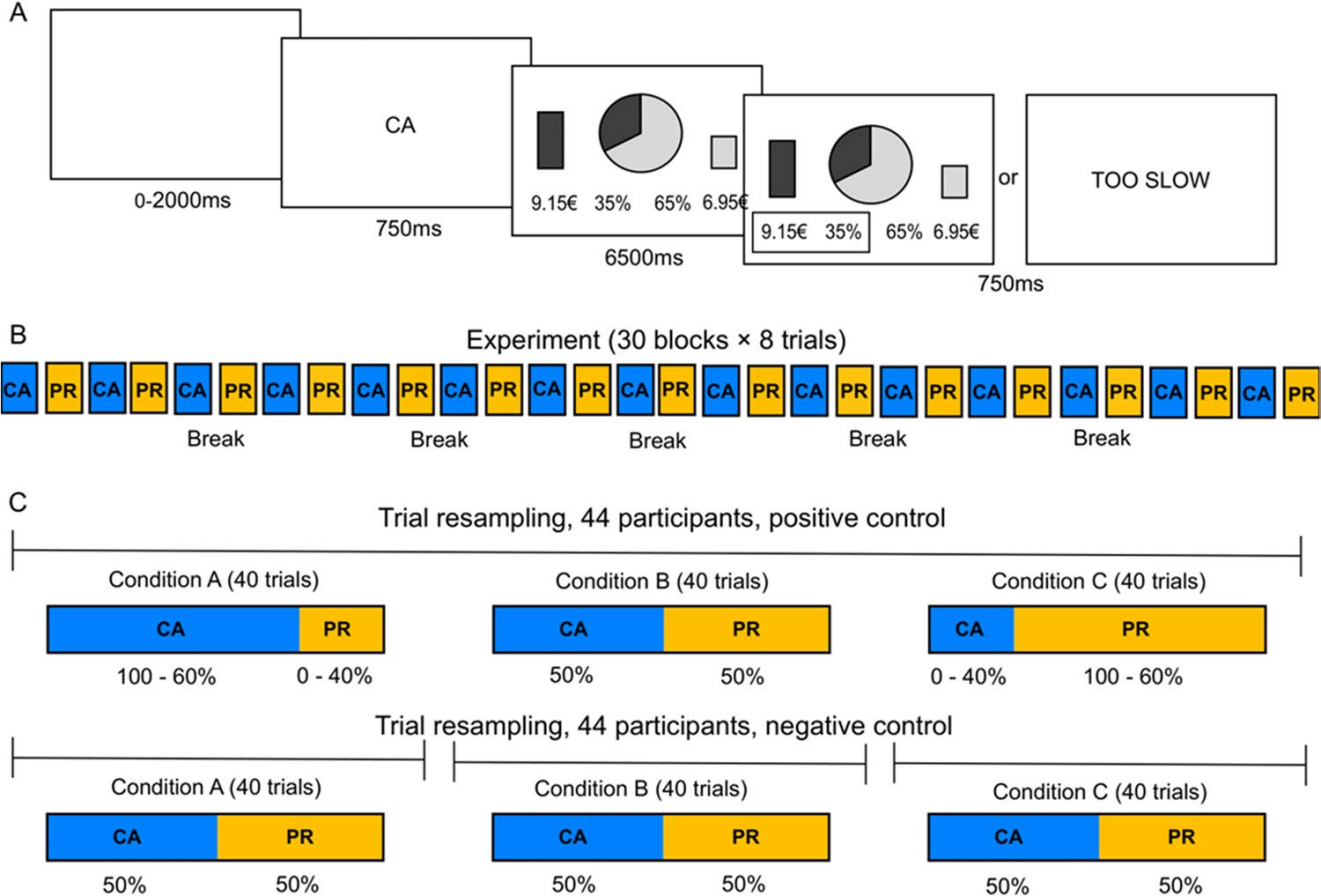
**A** Behavioral task. Successive screenshots displayed during a given trial are illustrated from left to right, with durations in milliseconds. On each trial, following a variable jitter (0–2000 ms) and a cue (750 ms), participants had to choose between a risky (left: 35% chance of winning or losing €9.15) and a safe (right: 65% chance of winning or losing €6.95) lottery. Choice durations are fixed (6500 ms), and followed by a choice-confirmation screen, where the selected lottery is highlighted by a contour box; or followed by a ”TOO SLOW” feedback if no lottery is selected (750 ms). Altogether, each trial is 10,000 ms long. **B** Experimental design. In total, participants performed 240 trials, spread over 30 blocks (i.e., 30 blocks of 8 trials), and they were provided with a short break every five blocks. Within each participant, CA and PR conditions were alternated between blocks; and between participants, the order of CA and PR conditions was counterbalanced. **C** Resampling approach in Scenario 1. For each participant (44 in total), 40 trials were resampled from the CA and PR conditions to form three new conditions A, B, and C. In the positive scenario, condition A contains a high proportion of CA trials (ranging from 100% to 60%) and a low proportion of PR trials (ranging from 0% to 40%); condition B, an equal number of CA and PR trials (50%); and condition C, a low proportion of CA trials (ranging from 0% to 40%) and a high proportion of PR trials (ranging from 100% to 60%). This mimics a mixture of processes under CA and PR conditions. In the negative scenario, condition A, B, and C contain an equal number of CA and PR trials (50%). This mimics the absence of a mixture of processes. To ensure that conditions A, B, and C differed in the negative scenario, in condition A, CA and PR trials were resampled from the beginning of the experiment (first 33% of the data); in condition B, from the middle (second 33% of the data); and in condition C, from the end (last 33% of the data)

The task consisted of a repeated binary decision-making task involving probabilistic monetary outcomes (Fig. 3A). On each trial, participants had to choose between a safe (i.e., *p* > 50% of winning a certain amount of money *a*) and a risky (1 − *p* of winning a higher amount *A*) lottery. The probabilities of each lottery were presented as two complementary areas of a wheel of fortune, displayed on the middle of the screen, and the amounts as vertical bars of varying height, displayed on the left or right of the screen (depending on which side the corresponding lottery was presented). The lottery displayed on the left of the screen was colored in blue, and the lottery displayed on the right of the screen was yellow. The side of presentation (left or right) of the safe and risky lotteries was randomized across trials. Text describing exact probabilities and amounts of the lotteries was also presented at the bottom of the screen.

In the calculate (CA) condition, participants were instructed to calculate the expected value (EV) of the lotteries at stake (i.e., the product of probability and amount; e.g., *EV = p × a*, in the case of the safe lottery) and to select the lottery with the highest EV. In the preference (PR) condition, they were instructed to choose the lottery according to their own preference. Each trial was preceded by a cue (CA or PR, respectively) to remind participants of the current instruction. These instructions were associated with respective incentivization mechanisms (see below).

An experimental session consisted of 30 blocks of alternating conditions, and each block consisted of eight trials (Fig. 3B). A short break was provided after five blocks. The order of CA and PR conditions was counterbalanced between participants. Before the experimental session, participants experienced 16 trials with feedback so that they could familiarize themselves with the task. As feedback, the lottery they selected was either verified or executed, depending on whether they were instructed to calculate or to play the lottery, and the result was displayed. In case participants did not provide a choice within 6.5 seconds, a ”TOO SLOW” feedback was displayed instead. After the familiarization, the participants entered the fMRI scanner for the experimental session. The experimental session was identical to the training session, except that no feedback was provided (only the “TOO SLOW” feedback, in case of no choice). To incentivize compliance with the instructions, two of the participants’ choices—each one corresponding to one condition—were selected at the end of the experiment. If the selected choice from the CA condition was correct, participants received a bonus of €5. The selected lottery from the PR condition probabilistically determined a second bonus, the amount of which depended on the choice of lottery and a conversion rate. Conversion rates between experimental and real € were set such that participants could ultimately win up to €5.

### Scenario 1: Reference scenario

Because in this experiment participants were explicitly instructed and incentivized to either calculate the expected values of the lotteries at stake or to choose the lottery according to their own preference, we consider these two explicitly instructed and incentivized conditions to be the ground truth. With that in mind, we assume that the data from these conditions form the RT base distributions from which mixture distributions with various mixture proportions can be generated. In Scenario 1, we repeatedly resampled from these RT base distributions in three synthetic conditions to assess the probability of finding a fixed point when the resampled data constitute a mixture of varying proportions (sensitivity), versus the probability of finding a fixed point when the resampled data do not constitute a mixture, or a mixture of fixed proportion (specificity).

To this end, we resampled 40 trials from the two experimental conditions CA and PR to form three new *resampled* conditions A, B, and C (Fig. 3C). For positive scenarios—which contain a mixture of cognitive processes—condition A contained a high proportion of CA trials, ranging from 100% to 60% (with the remaining trials from the PR condition), condition B always consisted of an equal number of CA and PR trials, and condition C was always the reciprocal of condition A. In the negative scenarios—which do not contain a mixture of processes or a mixture of fixed proportions—all trials in resampled conditions A, B, and C, were resampled from the CA and PR conditions with equal probability. However, to ensure that the resampled conditions differed (for a detailed rationale, see Van Maanen et al., 2016), we resampled from the different parts of the experiment, such that all trials in resampled condition A were from the first 33% of the data, all trials in B were from the second 33% of the data, and all trials in C were from the last 33% of the data. Because participants sped up throughout the experiment (see Results), this led to a shift in the mean RT across the resampled conditions. We performed 1000 bootstrapping samples.

The fixed-point property was estimated with the fp package for R (available at https://cran.r-project.org/web/packages/fixedpointproperty/index.html). Following the procedure outlined in the Introduction, four steps were carried out: In step 1, we computed pairwise BFs using Bayesian pairwise *t*-tests on the resampled conditions (Rouder et al., 2009). In step 2, the RT distributions for each participant and each resampled condition were approximated using a Gaussian kernel-based density estimator (Silverman, 1986). In step 3, the RTs of the crossing points for each pair of density functions were computed. Given that we have three resampled conditions, we also have three pairs of density functions (e.g., A–B, B–C, A–C), and consequently, three crossing points per participant. In step 4, we computed the BFs for the presence of the fixed point using Bayesian analysis of variance (ANOVA) (Rouder et al., 2012).

### Scenario 2: Time-on-task effects

Considering that we ultimately aim at gauging sensitivity and specificity of the fixed-point property test for real RT data, it is important that we mimic mixtures that may be susceptible to certain experimental factors (in addition to the mixture proportion), and which may subsequently affect the sensitivity and the specificity of the fixed-point property test. One factor that is often neglected and/or not explicitly analyzed, though often present in experiments, is the duration of the experiment itself. Over the course of an experiment behavior may change, for example, due to increased familiarity with the task or learning (Correa et al., 2018; Van Maanen et al., 2012), fatigue (Ratcliff & Van Dongen, 2009), or even boredom (Mittner et al., 2015). This potentially impacts the sensitivity of the fixed-point property analysis, as changes in the base distribution compromise the stability of the fixed point (Van Maanen et al., 2016).

**Fig. 4:**
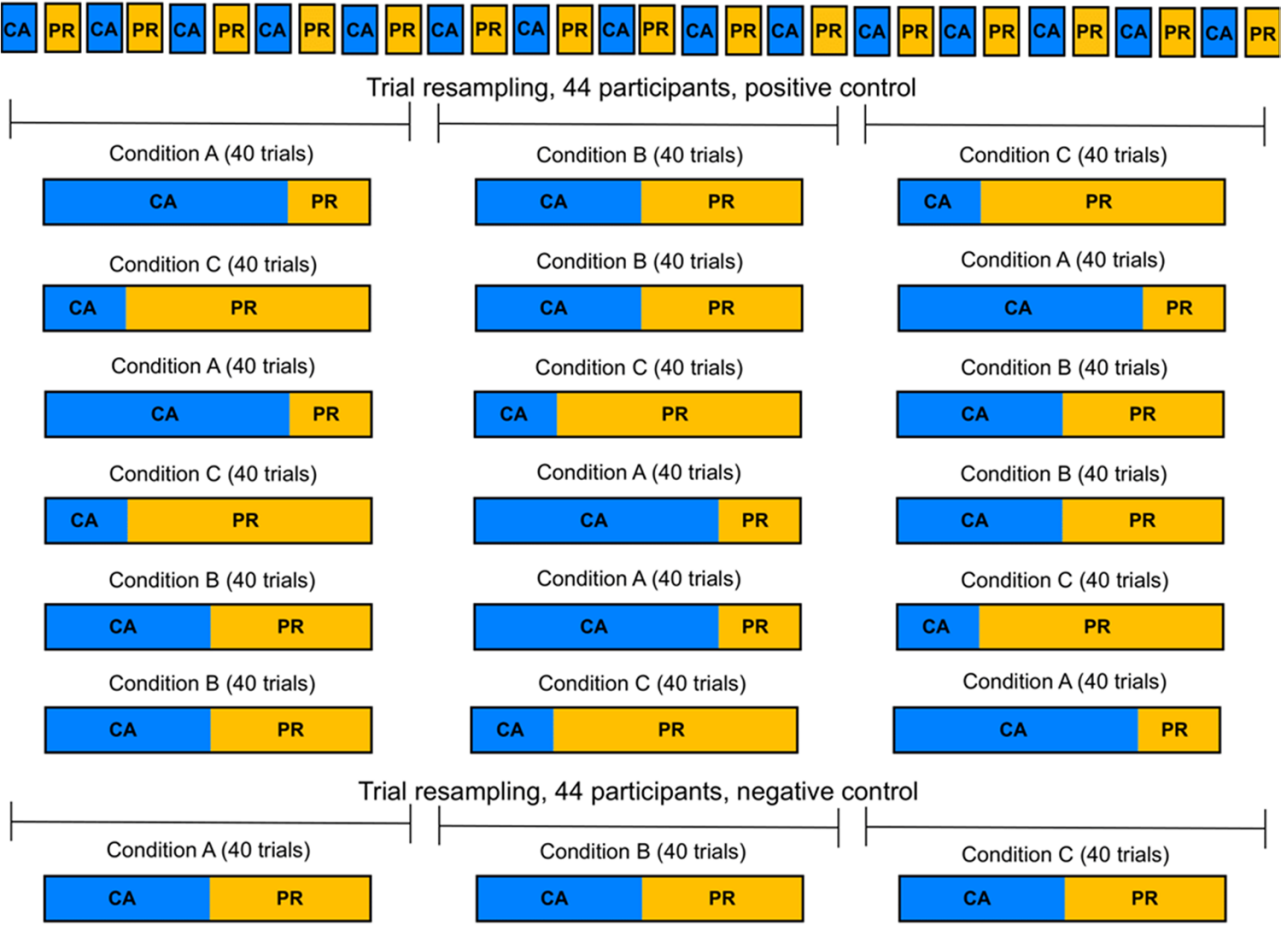
Resampling approach in Scenario 2. The positive and negative scenarios follow the same setup as Scenario 1, i.e., the positive scenario illustrates a change in the mixture proportion, and the negative scenario, no change in the mixture proportion. The difference in Scenario 2 stands on the way the CA and PR trials are resampled over the experiment to form conditions A, B, and C in the positive scenario—specifically, the probability that trials are resampled from the beginning, middle, or end of the experiment. A variation in this probability is to ensure a dependency between the different mixtures in conditions A, B, and C and the different modulations of the RT base distributions of CA and PR conditions across the different parts of the experiment. Given that the experiment is divided into three equals parts (beginning, middle, and end), there are six possible ways by which the mixtures in conditions A, B, and C can be arranged, i.e., six possible permutations

With this in mind, we generate different mixtures whose modulation of the two RT base distributions depends on the time on task. Specifically, in contrast to Scenario 1, we *also* varied the probability that trials were resampled from the beginning, middle, or end part of the data for the positive scenario. Because we chose to divide the experimental trials into three equal parts, there were six possible ways by which resampled conditions A, B, and C could be arranged over the three parts of the experiment (Fig. 4).

In all other respects, Scenario 2 follows the same setup as Scenario 1, i.e., a change in the mixture proportion for the positive scenarios (when the fixed point is present) and no change in the mixture proportion for the negative scenarios (when the fixed point is absent). In the positive scenarios, the mixture proportions are again systematically varied as in Scenario 1.

### Scenario 3: Sample size effects

**Fig. 5:**
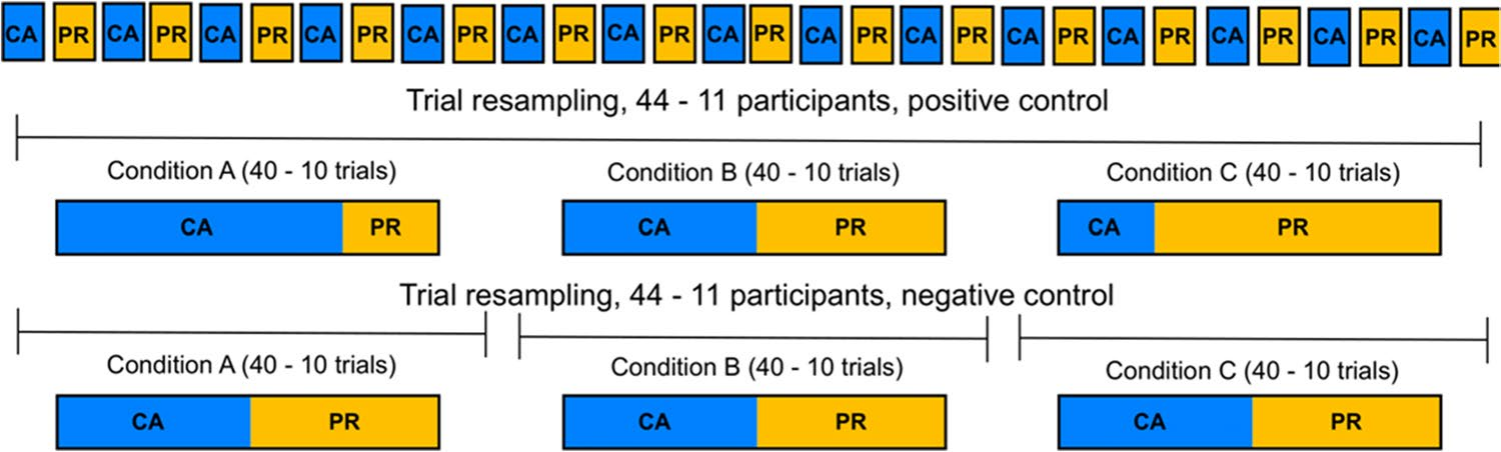
Resampling approach in Scenario 3. The positive and negative scenarios follow the same setup as Scenario 1, i.e., the positive scenario illustrates a change in the mixture proportion, and the negative scenario, no change in the mixture proportion. The difference in Scenario 3 stands on the number of participants and trials that are used and resampled to form conditions A, B, and C in the positive and negative scenarios. The number of participants is varied from 44 to 11 participants, and the number of resampled trials from 40 to 10 trials

In Scenario 3, we explored the lower limit of the sample size. This is important given the potential application of the fixed-point property test in domains where it is difficult or uncommon to collect large amounts of data, either in terms of participants or in terms of observations per participant. Thus, the distinctive feature of Scenario 3 is that we varied the number of both participants and observations in resampled conditions A, B, and C (Fig. 5). The number of participants was varied between 44 (the number of participants in the real experimental data) and 11, and the number of observations per participant was varied between 40 and 10 trials per condition. Again, Scenario 3 mimics Scenario 1 in all other respects, with the exception of the number of bootstrapping samples. We performed 10,000 bootstrapping samples to ensure that the results of Scenario 3 were stable, even when the number of participants and number of trials were extremely low.

### Receiver operating characteristic (ROC) curve analyses

To determine the sensitivity and specificity of the fixed-point property test using scenario analysis, we compute a ROC curve under the assumption that a fixed point is detected when the BF for the presence of a fixed point exceeds a certain criterion BF (which we will refer to as the fixed-point criterion). However, the detection of a fixed point depends on another criterion (which we refer to as the condition criterion) to assess whether the conditions initially differ (Van Maanen et al., 2016). The condition criterion safeguards against a situation where a detection of a fixed point may be wrongly inferred due to a lack of difference between RT distributions of the resampled conditions, rather than a true mixture proportion change. Detection or no detection of a fixed point therefore requires two sequential decision criteria. Firstly, with the condition criterion, we check whether there is a difference in all pairwise RT distributions using Bayesian pairwise *t*-tests. Secondly, if all pairwise BFs exceed the condition criterion, we proceed with the fixed-point criterion, where we check whether a fixed point is more likely to be present than absent. If one or more of the pairwise BFs do not exceed the condition criterion, we do not proceed with the fixed-point criterion and consider the fixed point to be absent. Together, these decision criteria demarcate what are traditionally known as true positive rate (TPR), true negative rate (TNR), false positive rate (FPR), and false negative rate (FNR). We thus computed a *conditional ROC*, where the detection of a fixed point by the fixed-point criterion is conditional on a specific choice of the condition criterion.

Modeling sensitivity and specificity using a conditional ROC has a number of consequences. Firstly, chance performance of the fixed-point property test is not at 50% as in standard binary choice, but at 25%, reflecting that there are two sources of classification instead of one. Secondly, because the condition criterion rejects some cases before they are matched against the fixed-point criterion, the TPR and TNR from the fixed-point criterion do not sum to 1. Consequently, the ROC curve may not reach the theoretical extreme where both the TPR and the FNR are 100% (see also Rotello et al., 2004; Wixted, 2007, for similar proposals, but for the study of human memory). These aspects are important to consider when interpreting the results.

A typical application of ROC analysis is to compute the area under the ROC curve (AUC) to reflect the ability to disentangle positive and negative cases (Bradley, 1997; Hanley & McNeil, 1982). Because the ROCs of the fixed-point criterion that we report here are conditional on a specific choice of the condition criterion, the AUCs also need to reflect this for a fair assessment. Therefore, we only consider the area where the curve is actually defined, by dividing the ROC by the overall FPR of the classification. This way, the probability of a true positive result by the fixed-point criterion, conditional on the probability of a false positive result by the choice of the specific condition criterion, is computed. Scripts to calculate the conditional AUCs are also available at https://osf.io/9vs3y.

## Results

**Fig. 6:**
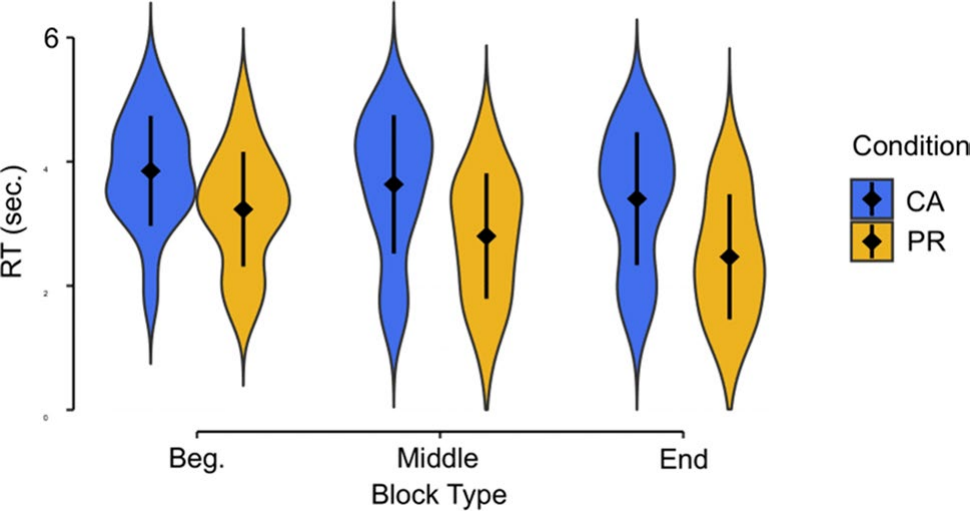
Observed RTs for the experimental conditions CA and PR in the beginning, middle, and end of the experiment. Each part of the experiment corresponds to one third of the total number of blocks (i.e., 10 blocks each part). Data points illustrate the mean of the median RTs, and error bars illustrate the standard deviation of the mean

To confirm that the two experimental manipulations that we depend on in the resampled scenarios are actually present in the data, we first investigated the effect of CA and PR conditions, as well as the effect of time on task (Fig. 6). A linear mixed-effect regression reveals that, overall, participants are faster in PR than CA (β_condition_ = −730, SE = 105, *p* < .001). Additionally, their speed increases overall throughout the experiment (β_time-on-task_ = −217 , SE = 38, *p* < .001), especially in the PR condition (β_time-on-task × condition_ = −153, SE = 23, *p* < .001). These results validate our approach, as the two different—instructed—strategies (i.e., CA and PR) indeed generate different base distributions of our behavioral variable of interest (i.e., RT). The significant effects of the time on task also substantiate our intuition that this factor might constitute an important confound if unaccounted for—a potential confound whose consequences are assessed in our Scenario 2.

### Scenario 1: Reference scenario

**Fig. 7:**
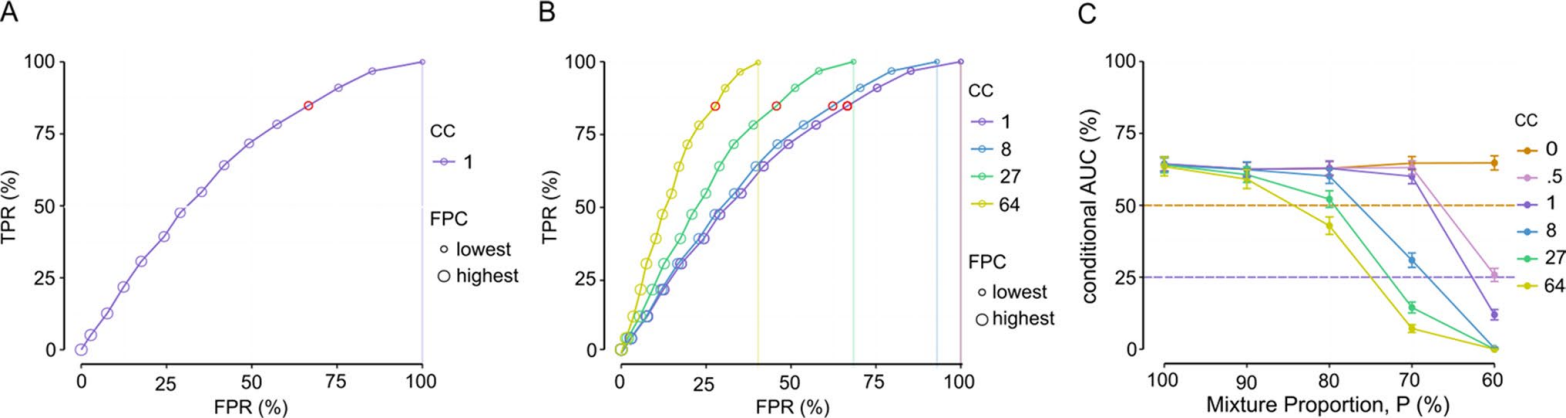
Results of Scenario 1. **A** Receiver operating characteristic (ROC) curve for the fixed-point criterion (FPC), conditional on a condition criterion (CC) of 1, with the mixture proportion P = 100%. The red circle illustrates FPC = 3. **B** Conditional ROC curves for various levels of CC, with the mixture proportion P = 100%. Conditional ROC curves with CC < 1 are not displayed here as they overlap. Vertical lines demarcate the area where the curve is actually defined. **C** Areas under the conditional ROC curves (conditional AUC) for various levels of CC and various mixture proportions. Data points and error bars illustrate the mean and 95% confidence intervals (CIs) of the conditional AUC over 1000 bootstrapping samples. Dashed lines illustrate chance performance for CC = 0 (in orange) and CC > 0 (in purple). Because a CC = 0 reduces the situation to unconditional AUC, chance performance is illustrated at 50% as in standard binary choice

Our first reference scenario features a mixture proportion P = 100% and a condition criterion of 1 (Fig. 7A). A mixture proportion P = 100% means that the three resampled conditions are respectively composed of 100% CA and 0% PR trials, 50% CA and 50% PR trials, and 100% CA and 0% PR trials. A condition criterion of 1 means that any amount of evidence in favor of a difference between the conditions is considered sufficient to carry on with the detection of a fixed point by the fixed-point criterion. Because such a condition criterion value is very permissive, no resampled cases are rejected at step 1, and the highest FPR (obtained for the lowest value of the fixed-point criterion, which is 0) can reach 100%. In this case, the conditional ROC curve is closed (i.e., there is a fixed-point criterion value for which TPR = 100% and FPR = 100%). When the value of the condition criterion increases, more resampled cases are rejected at step 1 due to a lack of difference between the resampled conditions, and therefore the highest FPR (obtained for the lowest value of the fixed-point criterion, which is again 0) mechanistically decreases. In those cases, the conditional ROC curves do not reach the theoretical extreme (Fig. 7B).

To get a sense of the sensitivity and specificity of the fixed-point test, we computed the areas under the conditional ROC curves (conditional AUC, cAUC) for different mixture proportions and for several values of the condition criterion (Fig. 7C). This systematic analysis reveals that, as the effect of the synthetic experimental manipulation on the mixture proportion decreases (i.e., as the mixture proportion *P* approaches 50%), the choice of a condition criterion has a large impact on the specificity of the fixed-point property (Van Maanen et al., 2016). The intuition behind this result is that, as the difference between the resampled conditions vanishes, the chances increase that the detection of a fixed point by the fixed-point criterion is stopped at step 1 due to the condition-difference test (especially for the most stringent values of condition criterion), mechanically inflating the FNR. Consequently, the TPR decreases and the AUC drops. This is illustrated in Fig. 7C, which shows the mean cAUC and 95% confidence intervals (CIs) over the resampled data (e.g., for CC = 64, cAUC_100_ = 63.5% ± 3.3, cAUC_90_ = 59.1% ± 3.3, cAUC_80_ = 42.9% ± 3, cAUC_70_ = 7.2% ± 1.4, cAUC_60_ = 0% ± 0). Here and throughout the paper, we interpret the CIs to understand changes in cAUC. This effect is naturally absent in the case where the condition criterion is set to 0, as the detection of a fixed point by the fixed-point criterion is not stopped at step 1, regardless of the mixture proportion. Consequently, the CIs do not reveal different mean cAUCs for a condition criterion of 0. Overall, these results reveal the importance of the choice of a condition criterion, as well as the effect size of the synthetic experimental manipulation on the mixture proportion.

### Scenario 2: Time-on-task effect

**Fig. 8:**
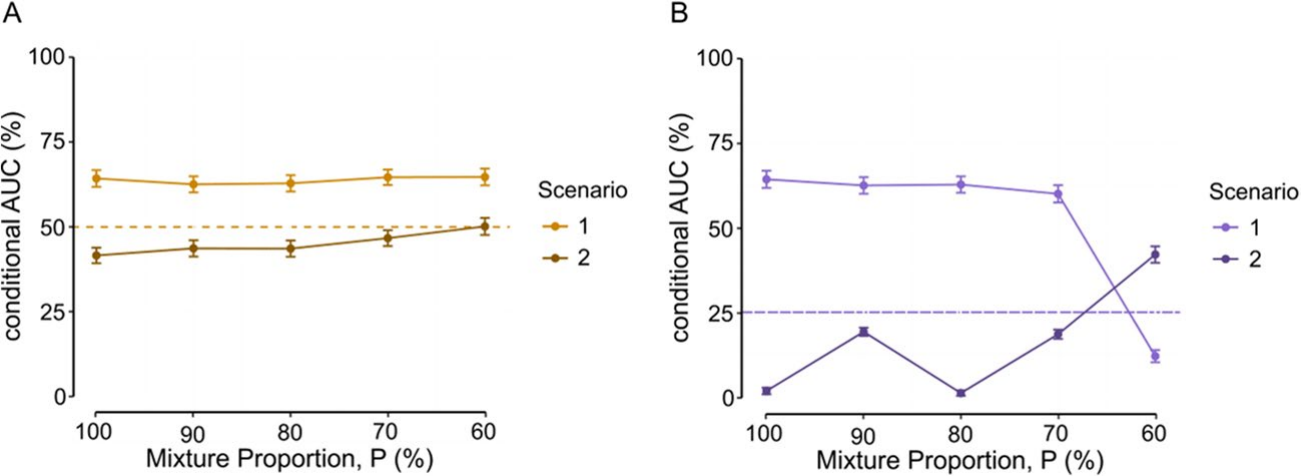
Results of Scenario 2. **A** AUC conditional on a CC of 0 and various mixture proportions. **B** AUC conditional on a CC of 1 and various mixture proportions. Dots and error bars for Scenario 2 illustrate the median of the mean and the mean of the 95% CIs of conditional AUC over all six arrangements (i.e., all six possible ways by which the resampled conditions can be arranged), each arrangement with 1000 bootstrapping samples. For comparison purposes, conditional AUC of Scenario 1 is added. Dashed lines illustrate chance performance

In Fig. 6, we observed an increase in RT speed over the course of the experiment. This observation substantiates our intuition that this might constitute an important confound if unaccounted for. In order to evaluate the consequences of this potential confound, we ran a scenario mimicking experimental designs that do not carefully distribute trials of the different conditions evenly throughout the experiment (Fig. 4). We computed the conditional AUC averaged across the six possible permutations of the resampled conditions (Scenario 2) and compared them with the conditional AUC from Scenario 1, which did control for the time on task in the different resampled conditions (Fig. 8). We first considered a situation of a permissive condition criterion (CC = 0). Note that a condition criterion of 0 means that no cases are excluded based on condition differences at step 1. This reduces the situation to unconditional AUC and chance performance to 50% as in standard binary choice. The resampled data under this situation revealed a severe drop in the AUC in Scenario 2 compared to Scenario 1 (Fig. 8A), with the AUC in Scenario 1 above chance performance and in Scenario 2 with no difference from chance. The intuition here is that the fixed-point test rejects the hypotheses that all crossing points are sampled from the same distribution at step 4, not because the mixture is absent but because it is occluded by the dependency generated between the mixture proportion change and the base distributions change across the experiment (Van Maanen et al., 2016). When we increased the stringency of the condition criterion (Fig. 8B, CC = 1), the number of false negatives increased to a greater degree, such that the cAUC dropped even more (e.g., cAUC_100_ = 1.7% ± 1, cAUC_90_ = 19.2% ± 1.2, cAUC_80_ = 1% ± 0.8) than when the condition criterion was set to 0 (panel A, e.g., cAUC_100_ = 41.6% ± 2.3, cAUC_90_ = 43.7% ± 2.4, cAUC_80_ = 43.6% ± 2.4). Interestingly, though, the severe drop in the more stringent condition criterion (Fig. 8B) is attenuated when the effect of the synthetic experimental manipulation on the mixture proportion decreases (i.e., cAUC_70_ = 18.5% ± 1.4, cAUC_60_ =42.1% ± 2.4). Above chance performance is observed in the cAUC when the effect is almost null (i.e., for cAUC_60_). This apparent improvement is, however, quite artificial, as it indicates that a larger number of resampled cases pass the stringent condition criterion at step 1, only to be rejected as false negatives later at step 4, inflating the conditional AUC. Overall, these results again emphasize the importance of the choice of a condition criterion, and the importance of carefully designing an experiment when one considers using the fixed-point property, as some apparently trivial details like the time on task can generate important confounds.

### Scenario 3: Sample size effects

**Fig. 9:**
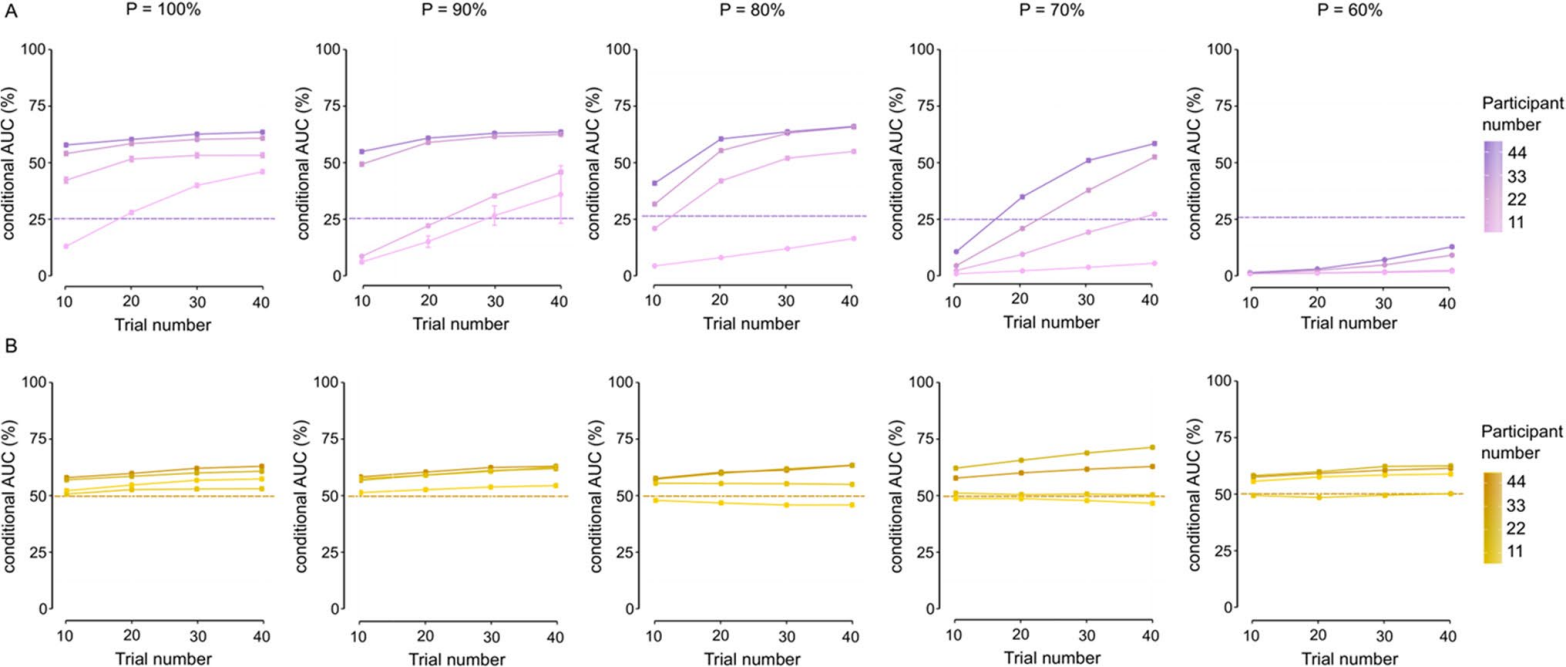
Results of Scenario 3. **A** AUC conditional on a CC of 1 and various mixture proportions. **B** AUC conditional on a CC of 0 and various mixture proportions. Dots and error bars illustrate the mean and 95% confidence intervals (CIs) of conditional AUC over 10,000 bootstrapping samples. Dashed lines illustrate chance performance

In this final section, we evaluate how the statistical power associated with the experimental design (i.e., number of trials and number of participants) impacts the sensitivity and specificity of the fixed-point test. To do so, we first compute the conditional AUC for different mixture proportions and a standard condition criterion of 1 (Fig. 9A). As could be intuited ex ante, the conditional AUC is an increasing function of both participant and trial numbers, regardless of the effect of the synthetic experimental manipulation (i.e., of the mixture proportion)—in other words, the specificity and sensitivity of the fixed-point test generally increase as the number of participants and the number of trials increase, and they decrease as the number of participants and the number of trials decrease. The highest cAUC that we obtained was 63.3% ± 0.8. This is significantly above chance performance, considering chance performance of 25%. This AUC or a comparable value was obtained for mixture proportions of 80% and higher, and for 33 participants or higher. The observation that there is no further increase in the cAUC when the number of participants increases beyond 33 suggests that a sample size of 33 is reasonable. However, the general trend in the AUCs does suggest that larger trial numbers are preferred—but see Rouder and Haaf (2018), where larger numbers of participants seem to be preferred. To gain a finer understanding of this result, we recomputed the AUC with a condition criterion of 0, which reduces the situation to unconditional AUC and chance performance to 50% as in standard binary choice (Fig. 9B). In this case, the effects of the number of trials and participants on the AUC are significantly attenuated, suggesting that the main effect—i.e., increase or decrease—of the statistical power operates through the condition criterion. These results emphasize once again the importance of the choice of a condition criterion, especially when there is a restriction on the number of participants and/or trials per participant.

## Discussion

The fixed-point property is a useful property of distributions of measured behavior for which a mixture of two cognitive processes is hypothesized (Falmagne, 1968). Although researchers have applied fixed-point property analysis to identify mixture distributions in their data (Brown et al., 2006; Couto et al., 2020; Grange, 2016; Katsimpokis et al., 2020; Poboka et al., 2014; Van Maanen, 2016), little is known about its ability to detect a mixture in the data when present (sensitivity of the fixed-point property) and to detect the absence of a mixture when absent (specificity of the fixed-point property). The novel contribution of this paper is the systematic investigation of the diagnostic ability of the fixed-point test under three different resampled scenarios, which mimic variations in the experimental design suspected to affect the sensitivity and specificity of the fixed-point test. This form of scenario analysis (Huss, 1988), which though widely applied in forecasting models (Sandmann et al., 2021; Xiao et al., 2019) is not well known in the domains of psychological measurement, yields a much broader application of the fixed-point test than the typical approach. Importantly, it also preserves important authentic features of data which are typically observed in real experiments while maintaining tight control over the properties of the resampled scenarios—for example, the true distribution in the data of each resampled scenario.

When cast in a signal detection framework, the conditional AUC in the various scenarios did not approach the ceiling, indicating that true fixed points in the data were not always detected, false fixed points were detected, or both. The highest average AUC that we found was 64%, and this was found when we used a very permissive condition criterion (i.e., CC = 0), so no cases were excluded based on condition differences. As soon as the condition criterion was higher, then detecting a fixed point became increasingly difficult, especially when the overlap between resampled distributions increased. This occurred because of changes in the mixture proportion (Scenario 1), covarying trends in the data (Scenario 2), and statistical power (Scenario 3).

Although the conditional AUCs were not particularly high in any configuration of the data, the sensitivity and specificity of the fixed-point test should be discussed and interpreted in the light of its dual criterion. Specifically, the choice of the condition criterion is important. When the condition criterion is set to 1 or higher, chance performance of the fixed-point property test is only 25%, reflecting two sources of classification—one based on the condition criterion and the other on the fixed-point criterion. This is substantially lower than the conditional AUCs that we report here, at least for some meaningful configurations of the data (i.e., scenarios). Consequently, a condition criterion of 0 is not always an optimal condition criterion. Putting this into perspective, a stricter condition criterion may actually be more optimal when the experimental conditions differ substantially (and this difference is not confounded with other experimental factors). This can for example be observed in the conditional AUCs of Scenario 1 for higher mixture proportions in relation to chance performance in Fig. 8A and B. Similarly, a stricter condition criterion may be more optimal when large amounts of data are possible, either in terms of participants or in terms of observations per participant, as for example was the case for the conditional AUCs in Fig. 9A and B.

Altogether, these results provide some important performance metrics to researchers aiming to apply the fixed-point property on their experimental data. In addition to the choice of the condition criterion, maximizing the effect size between experimental conditions (e.g., by optimizing experimental designs) and the sample size (e.g., by increasing the number of participants and trials) is particularly relevant for better performance of the fixed-point test. Although these research practices are already generally identified as good research practices (Ioannidis, 2005; Meyvis & van Osselaer, 2018; Simmons et al., 2011), there are still many methodological differences regarding these in the different domains. Taking the sample size as an example, both cognitive psychology and experimental economics encourage the collection of large amounts of data; however, while cognitive psychology does this in terms of both participants and observations per participant (Rouder & Haaf, 2018), experimental economics focuses in particular on the number of participants (Gruener, 2019). The illustrative example from the current manuscript thus emphasizes how these research practices should be fully considered when testing the fixed-point property, regardless of the research field. Regarding the choice of the condition criterion per se, there are no clear recommendations, as it greatly depends on those research practices. The general recommendation is that a very permissive condition criterion is not always an optimal condition criterion—i.e., a stricter condition criterion may be more optimal if the effect size between the experimental conditions and the sample size of the study are high. To make the choice of a condition criterion more systematic, however, a more concrete recommendation is that researchers engage on their own simulations, akin to parameter recovery and model identification exercises which are typically done in model-based analyses (Wilson & Collins, 2019).

Importantly, the fact that conditional AUCs were not particularly high in any configuration of the data could also be discussed and interpreted in light of the difficulty of the fixed-point test. Identifying a binary mixture of RT data is a notoriously difficult problem (Krajbich et al., 2015). This is because the detection of a mixture hinges on the estimation of the probability densities of the observed distributions, which are necessarily noisy samples. To tackle this, researchers have relied on multivariate data, such as including accuracy rates in addition to RTs (Archambeau et al., 2022; Molenaar et al., 2016; Visser, 2011). But this is not the case of the fixed-point test, which relies only on the univariate estimation of the RT densities. A second approach to tackle the difficulty in detecting binary mixtures is to make assumptions about the shape of the RT distribution (Molenaar et al., 2018). This enforces a theoretical model on the observed data, which researchers may not be prepared to do. The fixed-point property test provides a completely model-free method for detecting mixtures, which may come at the cost of lower accuracy. Considering this, an interesting analysis strategy could be the complementary use of model-free and model-based methods when investigating binary mixtures. The model-based approach may be more sensitive for small effects, but the model-free approach, such as fixed-point detection, allows for a corroboration of the assumptions underlying a model-based method.

In this data set, participants were explicitly instructed and incentivized to choose between lotteries according to two different strategies. This experimental task differs substantially from the tasks most often used in behavioral economics (Kirchler et al., 2017; Kocher & Sutter, 2006), where participants are subjected to time pressure or time constraints. The rationale behind these manipulations is that different strategies have different processing speeds (Evans, 2003; Sloman, 1996), so when participants choose under time pressure or time constraints, they use a faster strategy (Rubinstein, 2007). Although these manipulations have been proven valuable tools for the identification of different strategies in economic decision-making under risk (Spiliopoulos & Ortmann, 2018), they still face a number of challenges (Keren & Schul, 2009; Melnikoff & Bargh, 2018). Lack of precision in strategy specification is one of them. In fact, in the absence of explicit instructions, participants can use more than one faster strategy when subjected to these manipulations. We attempted to mitigate this problem by isolating specific strategies through instruction and incentivization. The validity of the task in this respect was further independently shown in a recent paper (Archambeau et al., 2022), which correctly identified the two instructed and incentivized strategies in the data (both RT and choices) using hidden Markov modeling (HMM) of the time series in the task. In case the HMM identified more than the two instructed and incentivized strategies, we could still use the fixed-point property, but only if our manipulation affected the proportion of the two instructed and incentivized strategies, as that would reduce the problem to a binary mixture in the end. If the experimental manipulation affected the proportion of other strategy(ies), however, then the fixed-point property would not apply, and more complex, model-driven analyses such as the HMM would be needed (Archambeau et al., 2022; Dutilh et al., 2011; Visser & Speekenbrink, 2014).

In conclusion, although the diagnostic ability of the fixed-point test has been revealed to be less than perfect, we have identified, through systematic investigations, which configurations of the data can improve its ability. Specifically, this includes an appropriate choice of a condition criterion, together with a maximization of the effect size—so that the experimental conditions differ substantially, and this difference is not confounded with other experimental factors—and a maximization of the sample size, in terms of both participants and observations per participant. We emphasize that the decision of the condition criterion is up to the researcher, who must decide according to the experimental design and sample size of the study, and ideally, based on their own simulations. We further argue in favor of the fixed-point test as a valid tool to detect different strategies, given its nature—i.e., dual criterion—and difficulty, as well as in favor of the lottery task in which the test was analyzed so as to detect different strategies in economic decision-making under risk.
